# Crystal structure and conformational analysis of 2-hy­droxy-3-(2-methyl­prop-1-en-1-yl)naphthalene-1,4-dione

**DOI:** 10.1107/S2056989015024755

**Published:** 2016-01-16

**Authors:** Sannyele Alcantara Emiliano, Sheyla Welma Duarte Silva, Mariano Alves Pereira, Valeria R.dos Santos Malta, Tatiane Luciano Balliano

**Affiliations:** a Institute of Chemistry and Biotechnology – IQB, Federal University of Alagoas - UFAL, Maceio–Alagoas, Brazil

**Keywords:** crystal structure, naphtho­quinone derivative, mol­ecular conformation, hydrogen bonding

## Abstract

In the structure of the title compound, C_14_H_12_O_3_, the substituent side chain, in which the H atoms of both methyl groups are disordered over six equivalent sites, lies outside of the plane of the naphthalene­dione ring. The ring-to-chain C—C—C—C torsion angles are 50.7 (3), −176.6 (2) and 4.9 (4)°. An intra­molecular meth­yl–hy­droxy C—H⋯O hydrogen bond is present. In the crystal, mol­ecules are primarily connected by inter­molecular O—H⋯O hydrogen bonds, forming a centrosymmetric cyclic dimer motif [graph set *R*_2_^2^(10)]. Also present is a weak inter­molecular C—H⋯O hydrogen bond linking the dimers and a weak π–π ring inter­action [ring centroid separation = 3.7862 (13) Å], giving layers parallel to (10-3).

## Abstract 2

In the structure of the naphtho­quinone derivative 2-hy­droxy-3-(2-methyl­prop-1-en-1-yl)naphthalene-1,4-dione, the mol­ecules form a centrosymmetric cyclic dimer through inter­molecular O—H⋯O hydrogen bonds which, together with inter­molecular C—H⋯O hydrogen bonds and weak π–π ring inter­actions, give rise to an overall two-dimensional structure.

## Chemical context   

Naphtho­quinone compounds exhibit several biological activities, being utilized for the treatment of parasitic diseases (Salas *et al.*, 2008 ▸) some types of cancer (Tonholo *et al.*, 1998 ▸) and cardiovascular disease (Silva & Torres, 2013 ▸). The compound in this study, 2-hy­droxy-3-(2-metilprop-1-enol)naphthalene-1,4-dione, C_14_H_12_O_3_, is a naphthoquinone deriv­ative and the structure is reported herein.
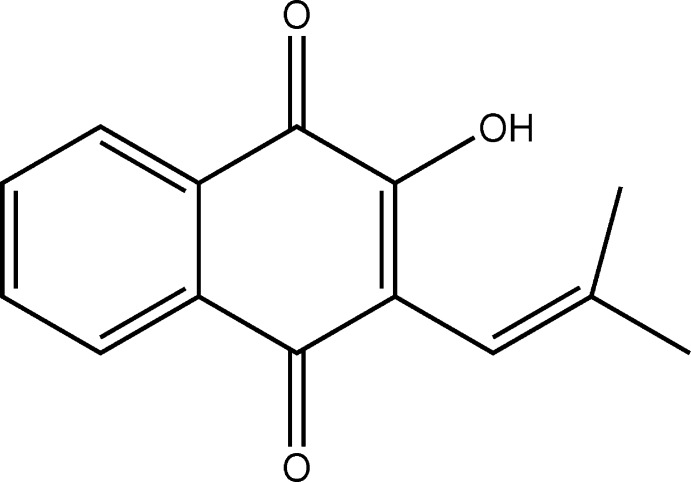


## Structural commentary   

**Figure 1:**
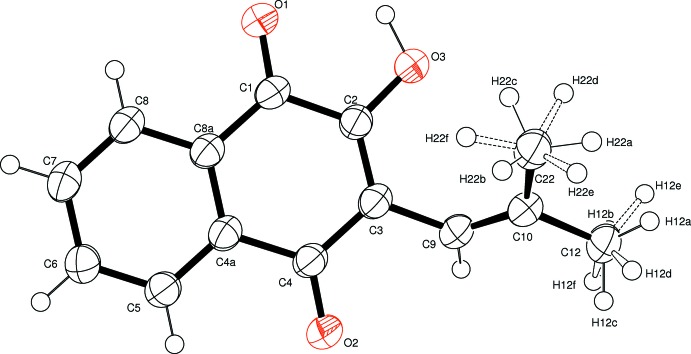
Mol­ecular conformation and atom-numbering scheme, with non-H atoms drawn at the 50% probability level. The H atoms of the rotationally disordered methyl groups are shown as six equivalent half-occupancy sites.

The mol­ecular structure of the title compound is shown in Fig. 1 ▸. In this structure the side chain is rotated out of the plane of the naphthalene­dione ring, with torsion angles C2—C3—C9—C10, C3—C9—C10—C12 and C3—C9—C10—C22 of 50.7 (3), −176.6 (2) and 4.9 (4)°, respectively. Present also in the mol­ecule is an intra­molecular methyl C22⋯O3 [2.959 (3) Å; see Table 1 ▸] and a short O3⋯O1 contact [2.665 (2) Å]. When compared with other analogous structures in the literature, *e.g.* 2-chloro-3-(4-chloro­benzamido)-1,4-naphtho­quinone (Brandy *et al.*, 2009 ▸), it is observed that the title compound has similar conformational features with respect to the side chain, which lies out of the naphtho­quinone plane.

## Supra­molecular features   

**Figure 2:**
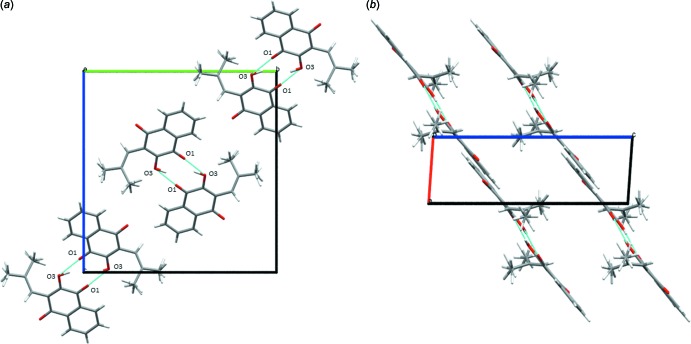
The centrosymmetric dimers formed from the O3—H⋯O1^i^ hydrogen bonds, viewed (*a*) along *a* and (*b*) along *b*. For symmetry code (i), see Table 1 ▸.

In the crystal, the mol­ecules are connected by classic inter­molecular O3—H⋯O1^i^ hydrogen bonds (Table 1 ▸), forming a centrosymmetric cyclic dimer [graph set 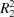
(10)] (Bernstein *et al.*, 1995 ▸) (Fig. 2 ▸ *a*). Also present in the structure is a weak inter­molecular C7—H⋯O2^ii^ hydrogen bond [3.339 (3) Å], linking the dimers and a weak π–π ring inter­action between the benzene and quinone ring moieties of the parent ring system [ring centroid separation *Cg⋯*Cg** ^iii^ = 3.7862 (13) Å; symmetry code: (iii) *x* + 1, *y*, *z*], giving layers parallel to (10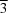) (Figs. 2 ▸ *b* and 3 ▸).

## Database survey   

A search of the Cambridge Structural Database (Groom & Allen, 2014 ▸) revealed the presence of 40 structures containing the 2-hy­droxy­naphthalene-1,4-dione core moiety. There were 787 structures which possess the naphthalene-1,4-dione moiety. There are structures similar to the title compound, whichvary depending on the oxidant used in the synthesis.

## Synthesis and crystallization   

The compound was obtained through to the lapachol oxidation product as can be seen in the scheme below (Hooker, 1936 ▸). The sample was subjected to an ethyl acetate solution at 301 K for crystallization.
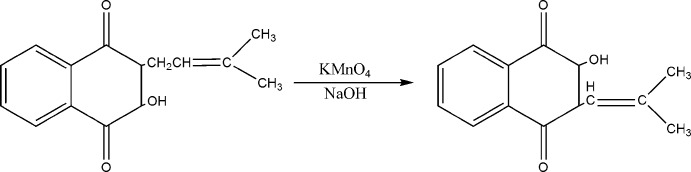


## Refinement   

Crystal data, data collection and structure refinement details are summarized in Table 2 ▸. The O3-bound H atom was located in a difference Fourier map and was freely refined. The remaining H atoms were positioned geometrically with aromatic C—H = 0.93 Å and *U*_iso_(H) = 1.2*U*_eq_(C). Rotational disorder was identified in the hydrogen atoms of the methyl carbon atoms C12 and C22 and these were included in the refinement over six equivalent 60° sites with 50% occupation, with C—H = 0.96 Å and *U*_iso_(H) = 1.5*U*_eq_(C).

## Supplementary Material

Crystal structure: contains datablock(s) I. DOI: 10.1107/S2056989015024755/zs2357sup1.cif

Structure factors: contains datablock(s) I. DOI: 10.1107/S2056989015024755/zs2357Isup2.hkl

Supporting information file. DOI: 10.1107/S2056989015024755/zs2357Isup3.cml

CCDC reference: 1444109

Additional supporting information:  crystallographic information; 3D view; checkCIF report

## Figures and Tables

**Figure 3:**
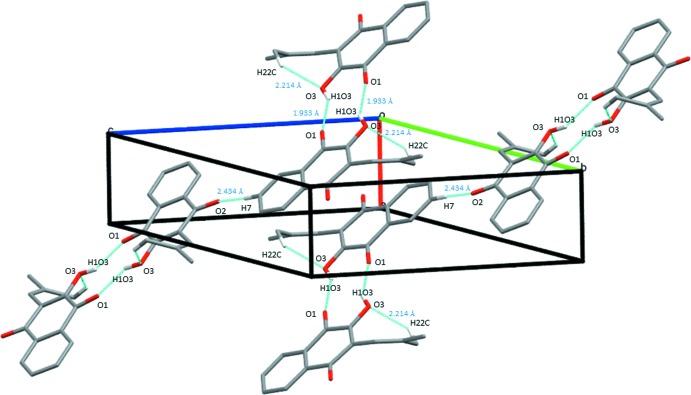
The crystal packing in the unit cell, showing intra- and inter­molecular inter­actions as dashed lines.

**Table 1:** Hydrogen-bond geometry (Å, °)

*D*—H⋯*A*	*D*—H	H⋯*A*	*D*⋯*A*	*D*—H⋯*A*
O3—H1*O*3⋯O1^i^	0.97 (3)	1.93 (3)	2.770 (2)	143 (3)
C7—H7⋯O2^ii^	0.93	2.43	3.339 (3)	164
C22—H22*C*⋯O3	0.96	2.21	2.959 (3)	134

Symmetry codes: (i) 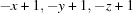; (ii) 
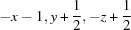
.

**Table 2:** Experimental details

Crystal data	
Chemical formula	C_14_H_12_O_3_
*M*_r_	228.24
Crystal system, space group	Monoclinic, *P*2_1_/*c*
Temperature (K)	293
*a*, *b*, *c* (Å)	4.3564 (2), 16.4069 (8), 15.8598 (7)
β (°)	94.793 (2)
*V* (Å^3^)	1129.62 (9)
*Z*	4
Radiation type	Mo *K*α
μ (mm^−1^)	0.09
Crystal size (mm)	0.14 × 0.11 × 0.10

Data collection	
Diffractometer	Nonius KappaCCD
No. of measured, independent and observed [*I* > 2σ(*I*)] reflections	4661, 2585, 1802
*R*_int_	0.041
(sin θ/λ)_max_ (Å^−1^)	0.650

Refinement	
*R*[*F* ^2^ > 2σ(*F* ^2^)], *wR*(*F* ^2^), *S*	0.061, 0.191, 1.03
No. of reflections	2585
No. of parameters	158
H-atom treatment	H atoms treated by a mixture of independent and constrained refinement
Δρ_max_, Δρ_min_ (e Å^−3^)	0.31, −0.30

Computer programs: *COLLECT* (Enraf–Nonius, 2001 ▸), *DENZO* and *SCALEPACK* (Otwinowski & Minor, 1997 ▸), *SHELXS97* and *SHELXL97* (Sheldrick, 2008 ▸), *ORTEP-3 for Windows* and *WinGX* (Farrugia, 2012 ▸), *Mercury* (Macrae *et al.*, 2008 ▸), *publCIF* (Westrip, 2010 ▸) and *PLATON* (Spek, 2009 ▸).

## supplementary crystallographic information

### Crystal data

**Table:**

C_14_H_12_O_3_	*F*(000) = 480
*M**_r_* = 228.24	*D*_x_ = 1.342 Mg m^−^^3^
Monoclinic, *P*2_1_/*c*	Mo *K*α radiation, λ = 0.71073 Å
Hall symbol: -P 2ybc	Cell parameters from 2659 reflections
*a* = 4.3564 (2) Å	θ = 1.0–27.5°
*b* = 16.4069 (8) Å	µ = 0.09 mm^−^^1^
*c* = 15.8598 (7) Å	*T* = 293 K
β = 94.793 (2)°	Block, red
*V* = 1129.62 (9) Å^3^	0.14 × 0.11 × 0.10 mm
*Z* = 4	

### Data collection

**Table:**

Nonius KappaCCD diffractometer	1802 reflections with *I* > 2σ(*I*)
Radiation source: Enraf-Nonius FR590	*R*_int_ = 0.041
Graphite monochromator	θ_max_ = 27.5°, θ_min_ = 2.6°
Detector resolution: 9 pixels mm^-1^	*h* = −5→5
CCD rotation images, thick slices scans	*k* = −19→21
4661 measured reflections	*l* = −20→20
2585 independent reflections	

### Refinement

**Table:**

Refinement on *F*^2^	Primary atom site location: structure-invariant direct methods
Least-squares matrix: full	Secondary atom site location: difference Fourier map
*R*[*F*^2^ > 2σ(*F*^2^)] = 0.061	Hydrogen site location: inferred from neighbouring sites
*wR*(*F*^2^) = 0.191	H atoms treated by a mixture of independent and constrained refinement
*S* = 1.03	*w* = 1/[σ^2^(*F*_o_^2^) + (0.0946*P*)^2^ + 0.4119*P*] where *P* = (*F*_o_^2^ + 2*F*_c_^2^)/3
2585 reflections	(Δ/σ)_max_ < 0.001
158 parameters	Δρ_max_ = 0.31 e Å^−^^3^
0 restraints	Δρ_min_ = −0.30 e Å^−^^3^

### Special details

**Table:**

Geometry. All s.u.'s (except the s.u. in the dihedral angle between two l.s. planes) are estimated using the full covariance matrix. The cell s.u.'s are taken into account individually in the estimation of s.u.'s in distances, angles and torsion angles; correlations between s.u.'s in cell parameters are only used when they are defined by crystal symmetry. An approximate (isotropic) treatment of cell s.u.'s is used for estimating s.u.'s involving l.s. planes.
Refinement. Refinement of *F*^2^ against ALL reflections. The weighted *R*-factor *wR* and goodness of fit *S* are based on *F*^2^, conventional *R*-factors *R* are based on *F*, with *F* set to zero for negative *F*^2^. The threshold expression of *F*^2^ > 2σ(*F*^2^) is used only for calculating *R*-factors(gt) *etc*. and is not relevant to the choice of reflections for refinement. *R*-factors based on *F*^2^ are statistically about twice as large as those based on *F*, and *R*- factors based on ALL data will be even larger.

### Fractional atomic coordinates and isotropic or equivalent isotropic displacement parameters (Å^2^)

**Table:**

	*x*	*y*	*z*	*U*_iso_*/*U*_eq_	Occ. (<1)
O3	0.3690 (4)	0.37038 (10)	0.48362 (10)	0.0407 (4)	
O1	0.2205 (4)	0.52143 (9)	0.43377 (9)	0.0404 (4)	
O2	−0.3406 (4)	0.27382 (9)	0.26721 (10)	0.0481 (5)	
C10	0.0940 (5)	0.19603 (13)	0.47932 (13)	0.0395 (5)	
C9	0.0749 (5)	0.23006 (12)	0.40272 (13)	0.0386 (5)	
H9	0.1126	0.1961	0.3578	0.046*	
H1O3	0.448 (7)	0.424 (2)	0.5005 (19)	0.073 (9)*	
C4A	−0.3114 (5)	0.41650 (12)	0.28476 (13)	0.0349 (5)	
C8A	−0.1689 (5)	0.48205 (13)	0.32860 (13)	0.0348 (5)	
C2	0.1441 (5)	0.38047 (12)	0.42115 (13)	0.0351 (5)	
C1	0.0733 (5)	0.46622 (12)	0.39675 (13)	0.0350 (5)	
C5	−0.5333 (5)	0.43122 (14)	0.21845 (13)	0.0400 (5)	
H5	−0.6268	0.3879	0.1885	0.048*	
C3	0.0007 (5)	0.31557 (12)	0.38155 (12)	0.0358 (5)	
C4	−0.2235 (5)	0.33077 (13)	0.30859 (13)	0.0369 (5)	
C6	−0.6151 (5)	0.51093 (14)	0.19709 (14)	0.0426 (5)	
H6	−0.7628	0.5207	0.1524	0.051*	
C8	−0.2532 (5)	0.56203 (13)	0.30691 (14)	0.0386 (5)	
H8	−0.1584	0.6056	0.3362	0.046*	
C7	−0.4789 (5)	0.57607 (13)	0.24159 (14)	0.0413 (5)	
H7	−0.5389	0.6291	0.2277	0.05*	
C12	0.1899 (6)	0.10858 (13)	0.49043 (15)	0.0475 (6)	
H12A	0.1919	0.0939	0.5491	0.071*	0.5
H12B	0.3923	0.1015	0.4718	0.071*	0.5
H12C	0.0468	0.0744	0.4575	0.071*	0.5
H12D	0.2288	0.086	0.4365	0.071*	0.5
H12E	0.0283	0.0784	0.5138	0.071*	0.5
H12F	0.3738	0.1055	0.5281	0.071*	0.5
C22	0.0189 (6)	0.23815 (14)	0.55869 (14)	0.0452 (6)	
H22A	0.0507	0.2013	0.6056	0.068*	0.5
H22B	−0.1923	0.2554	0.553	0.068*	0.5
H22C	0.1503	0.2848	0.5684	0.068*	0.5
H22D	−0.0449	0.2931	0.5457	0.068*	0.5
H22E	0.1981	0.2389	0.5983	0.068*	0.5
H22F	−0.1445	0.2095	0.583	0.068*	0.5

### Atomic displacement parameters (Å^2^)

**Table:**

	*U*^11^	*U*^22^	*U*^33^	*U*^12^	*U*^13^	*U*^23^
O3	0.0435 (9)	0.0323 (8)	0.0450 (9)	−0.0013 (6)	−0.0051 (6)	−0.0002 (7)
O1	0.0456 (9)	0.0318 (8)	0.0431 (8)	−0.0041 (6)	−0.0005 (6)	−0.0022 (6)
O2	0.0651 (11)	0.0308 (8)	0.0460 (9)	−0.0033 (7)	−0.0096 (7)	−0.0026 (7)
C10	0.0438 (12)	0.0302 (10)	0.0439 (12)	−0.0025 (8)	−0.0013 (9)	−0.0004 (9)
C9	0.0462 (12)	0.0282 (10)	0.0409 (11)	0.0009 (9)	0.0011 (9)	−0.0027 (9)
C4A	0.0422 (11)	0.0292 (10)	0.0340 (10)	−0.0011 (8)	0.0061 (8)	0.0012 (8)
C8A	0.0398 (11)	0.0305 (11)	0.0346 (10)	−0.0016 (8)	0.0057 (8)	−0.0001 (8)
C2	0.0384 (11)	0.0314 (11)	0.0355 (10)	0.0003 (8)	0.0035 (8)	0.0009 (8)
C1	0.0398 (11)	0.0288 (10)	0.0367 (10)	−0.0019 (8)	0.0055 (8)	−0.0034 (8)
C5	0.0486 (13)	0.0343 (11)	0.0368 (11)	−0.0025 (9)	0.0015 (9)	−0.0004 (9)
C3	0.0431 (11)	0.0292 (10)	0.0355 (10)	−0.0006 (8)	0.0064 (8)	−0.0006 (8)
C4	0.0453 (12)	0.0299 (10)	0.0357 (11)	−0.0025 (9)	0.0040 (9)	−0.0011 (8)
C6	0.0500 (13)	0.0383 (12)	0.0391 (11)	0.0013 (9)	0.0003 (9)	0.0039 (9)
C8	0.0460 (12)	0.0293 (10)	0.0410 (11)	−0.0005 (8)	0.0062 (9)	−0.0002 (8)
C7	0.0493 (12)	0.0312 (11)	0.0436 (11)	0.0027 (9)	0.0060 (9)	0.0055 (9)
C12	0.0651 (15)	0.0315 (11)	0.0447 (12)	0.0020 (10)	−0.0024 (10)	0.0011 (9)
C22	0.0587 (14)	0.0346 (11)	0.0420 (12)	0.0006 (10)	0.0033 (10)	0.0011 (9)

### Geometric parameters (Å, º)

**Table:**

O3—C2	1.344 (3)	C3—C4	1.472 (3)
O3—H1O3	0.97 (4)	C6—C7	1.387 (3)
O1—C1	1.230 (2)	C6—H6	0.93
O2—C4	1.228 (2)	C8—C7	1.387 (3)
C10—C9	1.333 (3)	C8—H8	0.93
C10—C22	1.496 (3)	C7—H7	0.93
C10—C12	1.501 (3)	C12—H12A	0.96
C9—C3	1.472 (3)	C12—H12B	0.96
C9—H9	0.93	C12—H12C	0.96
C4A—C5	1.389 (3)	C12—H12D	0.96
C4A—C8A	1.398 (3)	C12—H12E	0.96
C4A—C4	1.498 (3)	C12—H12F	0.96
C8A—C8	1.398 (3)	C22—H22A	0.96
C8A—C1	1.469 (3)	C22—H22B	0.96
C2—C3	1.361 (3)	C22—H22C	0.96
C2—C1	1.485 (3)	C22—H22D	0.96
C5—C6	1.390 (3)	C22—H22E	0.96
C5—H5	0.93	C22—H22F	0.96
			
C2—O3—H1O3	108.3 (18)	C10—C12—H12C	109.5
C9—C10—C22	124.9 (2)	H12A—C12—H12C	109.5
C9—C10—C12	120.2 (2)	H12B—C12—H12C	109.5
C22—C10—C12	114.91 (19)	C10—C12—H12D	109.5
C10—C9—C3	127.1 (2)	H12A—C12—H12D	141.1
C10—C9—H9	116.5	H12B—C12—H12D	56.3
C3—C9—H9	116.5	H12C—C12—H12D	56.3
C5—C4A—C8A	119.67 (19)	C10—C12—H12E	109.5
C5—C4A—C4	120.09 (19)	H12A—C12—H12E	56.3
C8A—C4A—C4	120.23 (18)	H12B—C12—H12E	141.1
C4A—C8A—C8	120.21 (19)	H12C—C12—H12E	56.3
C4A—C8A—C1	119.46 (19)	H12D—C12—H12E	109.5
C8—C8A—C1	120.32 (19)	C10—C12—H12F	109.5
O3—C2—C3	121.45 (19)	H12A—C12—H12F	56.3
O3—C2—C1	115.56 (18)	H12B—C12—H12F	56.3
C3—C2—C1	122.95 (19)	H12C—C12—H12F	141.1
O1—C1—C8A	122.31 (19)	H12D—C12—H12F	109.5
O1—C1—C2	119.00 (18)	H12E—C12—H12F	109.5
C8A—C1—C2	118.68 (18)	C10—C22—H22A	109.5
C4A—C5—C6	119.8 (2)	C10—C22—H22B	109.5
C4A—C5—H5	120.1	H22A—C22—H22B	109.5
C6—C5—H5	120.1	C10—C22—H22C	109.5
C2—C3—C4	118.61 (19)	H22A—C22—H22C	109.5
C2—C3—C9	123.83 (19)	H22B—C22—H22C	109.5
C4—C3—C9	117.35 (18)	C10—C22—H22D	109.5
O2—C4—C3	120.66 (19)	H22A—C22—H22D	141.1
O2—C4—C4A	119.58 (18)	H22B—C22—H22D	56.3
C3—C4—C4A	119.76 (18)	H22C—C22—H22D	56.3
C7—C6—C5	120.7 (2)	C10—C22—H22E	109.5
C7—C6—H6	119.6	H22A—C22—H22E	56.3
C5—C6—H6	119.6	H22B—C22—H22E	141.1
C7—C8—C8A	119.7 (2)	H22C—C22—H22E	56.3
C7—C8—H8	120.2	H22D—C22—H22E	109.5
C8A—C8—H8	120.2	C10—C22—H22F	109.5
C6—C7—C8	119.9 (2)	H22A—C22—H22F	56.3
C6—C7—H7	120	H22B—C22—H22F	56.3
C8—C7—H7	120	H22C—C22—H22F	141.1
C10—C12—H12A	109.5	H22D—C22—H22F	109.5
C10—C12—H12B	109.5	H22E—C22—H22F	109.5
H12A—C12—H12B	109.5		
			
O1—C1—C2—O3	0.2 (3)	O2—C4—C4A—C5	2.6 (3)
O1—C1—C2—C3	−177.5 (2)	O2—C4—C4A—C8A	−176.8 (2)
C8A—C1—C2—O3	179.69 (19)	C3—C4—C4A—C5	−177.0 (2)
C8A—C1—C2—C3	2.0 (3)	C3—C4—C4A—C8A	3.5 (3)
O1—C1—C8A—C4A	175.2 (2)	C4—C4A—C5—C6	179.6 (2)
O1—C1—C8A—C8	−3.9 (3)	C8A—C4A—C5—C6	−1.0 (3)
C2—C1—C8A—C4A	−4.3 (3)	C4—C4A—C8A—C1	1.6 (3)
C2—C1—C8A—C8	176.6 (2)	C4—C4A—C8A—C8	−179.4 (2)
O3—C2—C3—C4	−174.50 (19)	C5—C4A—C8A—C1	−177.9 (2)
O3—C2—C3—C9	0.2 (3)	C5—C4A—C8A—C8	1.2 (3)
C1—C2—C3—C4	3.1 (3)	C4A—C5—C6—C7	−0.4 (3)
C1—C2—C3—C9	177.8 (2)	C5—C6—C7—C8	1.5 (3)
C2—C3—C4—O2	174.5 (2)	C6—C7—C8—C8A	−1.3 (3)
C2—C3—C4—C4A	−5.9 (3)	C7—C8—C8A—C1	179.0 (2)
C9—C3—C4—O2	−0.5 (3)	C7—C8—C8A—C4A	−0.1 (3)
C9—C3—C4—C4A	179.15 (19)	C3—C9—C10—C12	−176.6 (2)
C2—C3—C9—C10	50.7 (3)	C3—C9—C10—C22	4.9 (4)
C4—C3—C9—C10	−134.6 (2)		

### Hydrogen-bond geometry (Å, º)

**Table:**

*D*—H···*A*	*D*—H	H···*A*	*D*···*A*	*D*—H···*A*
O3—H1*O*3···O1^i^	0.97 (3)	1.93 (3)	2.770 (2)	143 (3)
C7—H7···O2^ii^	0.93	2.43	3.339 (3)	164
C22—H22*C*···O3	0.96	2.21	2.959 (3)	134

Symmetry codes: (i) −*x*+1, −*y*+1, −*z*+1; (ii) −*x*−1, *y*+1/2, −*z*+1/2.
